# Factors influencing radiation therapy student clinical placement satisfaction

**DOI:** 10.1002/jmrs.41

**Published:** 2014-01-28

**Authors:** Pete Bridge, Mary-Ann Carmichael

**Affiliations:** School of Clinical Sciences, Queensland University of TechnologyBrisbane, Australia

**Keywords:** Placement, radiation therapy, satisfaction, student

## Abstract

**Introduction:** Radiation therapy students at Queensland University of Technology (QUT) attend clinical placements at five different clinical departments with varying resources and support strategies. This study aimed to determine the relative availability and perceived importance of different factors affecting student support while on clinical placement. The purpose of the research was to inform development of future support mechanisms to enhance radiation therapy students’ experience on clinical placement.

**Methods:** This study used anonymous Likert-style surveys to gather data from years 1 and 2 radiation therapy students from QUT and clinical educators from Queensland relating to availability and importance of support mechanisms during clinical placements in a semester.

**Results:** The study findings demonstrated student satisfaction with clinical support and suggested that level of support on placement influenced student employment choices. Staff support was perceived as more important than physical resources; particularly access to a named mentor, a clinical educator and weekly formative feedback. Both students and educators highlighted the impact of time pressures.

**Conclusions:** The support offered to radiation therapy students by clinical staff is more highly valued than physical resources or models of placement support. Protected time and acknowledgement of the importance of clinical education roles are both invaluable. Joint investment in mentor support by both universities and clinical departments is crucial for facilitation of effective clinical learning.

## Introduction

Students undertaking the 3-year Bachelor of Radiation Therapy course at Queensland University of Technology (QUT) undertake 25 weeks of clinical placement throughout their training. These weeks are spread over five individual blocks and are aimed at facilitating development and assessment of clinical competencies as well as consolidating academic knowledge. Clinical placements are undertaken at a range of departments primarily across the State but also interstate. This range of clinical placements can lead to a variety of different experiences including different technology and equipment, differing practices and staffing models. Factors affecting this include the size of department, the funding model (private or public) and staff engagement with student education. The range of placements is designed to provide students with the widest possible variety of clinical experience. This contrasts with alternative models^1^ where students are assigned to a clinical site and are placed there throughout their training. This has the advantage of allowing students to become very familiar with the local practices and clinical staff; they accordingly spend less time adapting themselves to new environments. A study of a similar model of placement in nursing^2^ suggested that the familiarity with protocols and context enhanced student preparation for practice. Contrasting this from a clinical skills development perspective, the advantage of using a variety of placements means that students learn to become more independent and flexible, exposing them to a wide range of different protocols, techniques and equipment. There is little evidence comparing these two models of placement; however, a relatively small-scale 2013 Monash study^3^ contrasting continuous and block placements for 17 student midwives concluded that type of placement model experienced made less of an impact than input from clinical staff. No matter which model of placement is used there are many potential sources of support for students on clinical placement that Moreland and Carnwell^4^ categorised as ‘practical’, ‘emotional’ and ‘academic’ when designing their seminal Learning Support Needs Questionnaire (LSNQ) to identify key requirements of nursing students. Academic support for radiation therapy students while on placement is provided by clinical educators as well as student mentors. There are two models of educator provision; a dedicated full-time educator (or team) or an educator working in the role in addition to a full clinical workload. Academic processes to help maximise learning include provision of weekly formative feedback, assignation of a named mentor and access to dedicated clinical teaching activities. There is variance in availability of these and this is mainly related to time available for clinical educators. Practical support concerns physical resources that enable study and given the wide variety of clinical department resources it is not surprising that some centres are able to provide increased access to the internet, dedicated study areas, teaching resources and in-service (staff education) sessions compared to others. It is not clear to what extent this bewildering array of resources influence student satisfaction with clinical placement and impact on their learning within the departments.

### Aims

This study aimed to determine the relative availability and perceived importance of different factors affecting student support while on clinical placement. The purpose of the research was to inform development of future support mechanisms to enhance radiation therapy students’ experience on clinical placement.

## Methods

Perceptions of clinical placement support were gathered from undergraduate years 1 and 2 radiation therapy students from QUT and clinical educators from Queensland using an anonymous Likert-style questionnaire delivered via the online virtual learning environment. The survey was designed according to the guidelines of Moreland and Carnwell^4^ as recently validated by Price et al.^5^ in their survey of pre-registration nurses. Thus, questions relating to both the performance (availability) of various aspects of clinical placement support and their importance were included in order to guide resource development. Open questions also sought comments concerning equity of provision (e.g. ‘How important do you think it is to have similar support at different sites?’) and potential improvements to clinical placement support (e.g. ‘How could educational support be improved at your placement site?’).

Data were collected over the course of semester 1 in 2013 during which students had been on clinical placement. Year 3 students were excluded due to pressures associated with their final placement. Data were pertinent to the last placement experienced only. A total of 45 students and 12 educators were invited to participate. Additional data were introduced from the regular student placement satisfaction surveys to establish the relationship between overall satisfaction and students’ wishes in relation to future employment at their site. Likert-style responses were analysed using descriptive statistics and correlation analysis, while comments were subjected to qualitative thematic analysis to determine the importance of various factors as addressed in the Discussion section.

Participation was anonymous and voluntary. Ethical approval for the study was provided by the QUT Human Research Ethics Committee as part of a larger ethical approval for course development initiatives.

## Results

Placement support questionnaires were completed by a total of 18 students drawn from both years 1 and year 2 students as well as six clinical educators. Response rates for students were 40% and 50% for educators. Year 1 students had just completed a 2-week block of placement and year 2 had completed a 5-week block after a previous 8 weeks of placement. The results of the surveys including the focus of the Likert questions are presented in Tables 1 and 2.

**Table 1:** Availability and importance of clinical support mechanisms.

		Availability						Importance					
Support mechanism	Role	1	2	3	4	5	?	1	2	3	4	5	?
Clinical educator access	Student	2	2	1	5	8	0	0	1	0	10	6	0
	Educator	0	0	0	3	3	1	1	0	0	2	3	0
Internet access	Student	0	5	5	4	1	2	3	5	6	2	1	1
	Educator	0	1	0	2	3	0	1	0	3	2	0	0
Dedicated study area	Student	1	2	5	5	5	0	2	3	6	4	3	0
	Educator	0	3	2	1	0	0	0	4	1	0	1	0
Teaching on site	Student	0	1	7	6	4	0	0	2	2	13	1	0
	Educator	0	1	1	2	2	0	0	0	2	3	1	0
Additional ‘homework’	Student	1	4	7	2	3	1	0	8	7	3	0	0
	Educator	1	0	3	1	1	0	0	3	3	0	0	0
In-service attendance	Student	1	0	0	1	10	6	0	0	3	6	3	6
	Educator	0	0	1	2	3	0	0	0	1	3	2	0
Named mentor	Student	1	1	3	8	5	0	0	0	0	10	8	0
	Educator	0	1	0	3	2	0	0	0	0	4	2	0
Weekly formative feedback	Student	1	0	2	8	7	0	0	0	1	9	8	0
	Educator	0	0	0	2	4	0	0	0	0	2	4	0

*Availability*: 1, never available; 2, rarely available; 3, occasionally available; 4, usually available; 5, always available. *Importance*: 1, not at all important; 2, not that important; 3, reasonably important; 4, very important; 5, essential. ‘?’: don't know.

**Table 2:** ‘How important do you think it is to have similar support at different clinical placement sites?’

	A wide variety is very important	Some variety is good	Makes no difference	Important	Absolutely essential	Don't know
Student	0	0	7	0	10	1
Educator	0	0	5	0	1	0

Placement satisfaction data were available from the same student group but by using a different tool as part of standard audit procedures; this elicited 34 responses. Students and educators generally agreed about availability and importance of most of the factors. There was some disagreement about internet access and dedicated study area provision. The implications of these findings are debated in the Discussion section. Students were asked to rate their overall satisfaction with their placements and these mainly indicated a high level of satisfaction. Correlation of this satisfaction with students’ scores relating to ‘availability’ of the different support mechanisms was performed using the Pearson product-moment correlation coefficient.^6^ There was a highly significant strong positive correlation (*r* = 0.655 and *p* = 0.003), confirming the role of placement support mechanisms in student satisfaction with their placement experience. Correlation of student satisfaction with desire to seek employment at the placement site was unsurprisingly strongly correlated (*r* = 0.77 and *p* < 0.0001)^6^ suggesting that a positive experience on placement improved the chances of students wishing to apply for employment at the same department in the future. Investment in improvement to student placement experience is highly recommended for departments that struggle to recruit.

## Discussion

Thematic analysis of the findings was conducted to determine the relative impact of placement support factors on student satisfaction. Common issues related to satisfaction, equity of support, mentoring and limitations of support.

### Student satisfaction

The two survey tools indicated a high level of student satisfaction with clinical placements and it was interesting to note that the quality of student experience was positively strongly correlated with availability of clinical support mechanisms. Furthermore, data from the student satisfaction tool indicated a strong positive correlation between student placement satisfaction and desirability of placement site as a future employment option. This result echoed findings from a 2009 study^7^ into undergraduate midwifery students’ career intentions where it was concluded that positive placement experience influenced future career decisions. Both these findings indicate the potential importance of placement support as an ongoing recruitment method.

### Similarity of support

It was clear from the findings that there was a considerable difference in the extent of clinical support resources found across the range of clinical departments. Analysis of the qualitative responses indicated that most students valued a consistent approach to clinical placement support, whereas clinical educators could see the value of both consistency and a range. Students appreciated knowing what to expect on arrival, using responses including ‘stability’, ‘familiarity’ and ‘consistency’. Most of their comments, however, related more to equity of performance and assessment standards rather than learning support. The overall perception was summed up by the responses:

‘learn most on clinical placements’ (S1)

‘positive support and environment really make the experience enjoyable and beneficial’ (S2)

Most of the clinical educators were also in favour of a consistent approach and also highlighted the value of equity in experiences and maintaining consistent expectations for the students. There were some comments, however, highlighting the value of different support approaches. It was felt that this promoted initiative and independent learning strategies as well as allowing students to question different practices. The difference between a consistent approach and a range of different support mechanisms is echoed by the different practices adopted by universities for their clinical education provision. Australian undergraduate radiation therapy courses adopt a model where students experience a variety of placements throughout their training, whereas elsewhere, as in the United Kingdom it is common to keep the students at the same department for all of their placements.^1^ The relative advantages of each model follow similar themes of a balance between consistency and familiarity for students and variety of experiences from a learning perspective. While staying at the same centre allows students to undertake a stable and familiar clinical experience, it does restrict the opportunities available depending on each department's resources and practices. With a ‘variety of placements’ model adopted within the course at QUT it could be postulated that the range of different clinical support mechanisms and resources is less of an issue than if students only visited the same department.

### Relative importance of support resources

Analysis of the student responses clearly demonstrated a difference between those resources and mechanisms that were commonly perceived as important and those that were less so. The important aspects were; clinical educator availability, teaching, regular formative feedback and provision of a named mentor. This was echoed within the themes elicited from the qualitative comments as discussed later. Clinical support resources that were perceived as less important included; internet access, dedicated study areas and setting of additional written work. This contrasts with the findings of a Nigerian study^8^ that gathered medical imaging student placement perceptions and recommended increased provision of information technology (IT) and library facilities. This difference may be due to differences between the two professional course pedagogical approaches or simply increased access to IT and online resources over the last 6 years. Educator comments suggested that a dedicated study area would be of value which initially appeared to contradict the student perception about study areas but comments demonstrated that this was related more to provision of feedback rather than additional study. It is clear from this study's findings that both students and educators valued people-related resources more than physical resources. It is not the physical environment as much as the interpersonal support that enhances learning on clinical placement.

### Mentorship

The most dramatic results in terms of students’ perceived importance of support mechanisms related to provision of a named mentor (45% considered it ‘absolutely essential’ and 55% found it ‘very important’) and availability of a clinical educator (35% ‘absolutely essential’, 59% ‘very important’ and one student ‘not at all’). These figures triangulated well with qualitative comments from the students regarding their suggestions for improvement. These consistently highlighted the value of mentorship while on clinical placement with typical comments including the following:

‘always someone there willing to help (not always the educator)’ (S3)

‘named “buddy” interested in 1 on 1 support’ (S4)

‘educators spend time with us throughout the week, not just at the end of the week’(S5)

Students value the input of dedicated clinical educators but are aware of the limitations imposed by time restrictions. There are different models of clinical educator support adopted throughout the placement departments with some departments employing a dedicated educator (or team) and some departments staffing the role on a voluntary basis with little time dedicated to education. The literature base for clinical educator models mainly relates to the field of nursing as summarised by Lambert and Glacken.^9^ There is a paucity of research into radiation therapy-specific clinical education models with only Doughty and Hodgson^1^ providing some valuable information contrasting UK academic and clinical-based support mechanisms; however, there was no evaluation of their efficacy provided. The findings from our Australian study had insufficient responses to determine if radiation therapy student satisfaction was influenced by clinical educator model and future work establishing this would be useful.

The value of named mentors for effective clinical education is consistently confirmed in the literature^5^,^10^,^11^; indeed in 2010 the UK Nursing and Midwifery Council stipulated that mentor allocation was compulsory.^12^ The results of this study strongly indicated the need for vigorous support for mentorship by the university. The resulting mentorship course and resources have been well received by mentors and informal student feedback indicates a tangible improvement in experience associated with mentors who have attended this. Successful clinical support is dependent on commitment and cooperation between academic and clinical departments.^13^ Myall et al.^10^ commented that a dependence on the ‘inherent goodwill of mentors’ should be replaced with a system of organisational support for mentorship and it is important that this is provided from both academic and clinical departments.

### Formative feedback

Feedback is widely accepted as an essential component of successful clinical learning^14^ so it is pleasing that both students and educators in this study highlighted the value of weekly formative feedback. Student survey results indicated the importance with 44% considering it ‘absolutely essential’, 50% ‘very important’ and 1 ‘reasonably important’. Despite the large time commitment of the formative feedback process 100% of students and educators reassuringly indicated that it was ‘usually’ or ‘always’ available. Educator comments highlighted the value of one-to-one interactions with mentors or educators as well as consistency of assessment and feedback mechanisms. It is pleasing to see that not only do the educators demonstrate a passion for regular feedback but that the students can see the value of it also.

### Time pressures

Clinical educators’ comments related more to the pressures of time.

‘Time is the biggest factor, being a clinical educator who has a full clinical load I have very limited time’ (E1)

There were commonly expressed themes relating to the challenges of finding sufficient time to spend with students and the resulting feelings of evident frustration and guilt. This confirms findings in the literature^10^,^15^ and strongly indicates the value of protected time for clinical educators. Both this study and evidence in the literature from the nursing profession confirm that the insufficient time available is further threatened as educators were usually required to perform additional clinical roles^13^,^16^,^17^ or prioritise graduate education over undergraduates. There were suggestions that the profile and relative value of the education role needed raising and this is again well supported by evidence from other professions.^16^,^18^

### Limitations

The low clinical educator response numbers frustrated attempts to determine themes. The study also relies strongly on self-reporting with the potential for inherent bias in responses due to a lack of objectivity about respondents’ roles. It is likely that the student responses are not subjected to bias, although there may be different levels of perception about clinical support related to the different year groups. Relatively small numbers of respondents prevented effective meta-analysis but an overview suggested that the 2-year groups had similar outlooks. It is also possible that assessment results and feedback may have biased responses; correlation with assessment results is impossible when responses are anonymous.

## Conclusions

The study findings confirmed that students were generally satisfied with the support they were able to access on clinical placement. There was a further suggestion that levels of support on placement had the potential to influence student desires for employment applications. Support mechanisms related to staff were perceived as more important than physical resources; particularly access to a named mentor, a clinical educator and weekly formative feedback. While students valued input from mentors, they were able to acknowledge the time-intensive aspects of mentorship and weekly feedback provision. Educators also stressed the impact of time pressures; strong support for clinical mentors from the academic team is highly recommended in order to equip clinical staff with tools to help provide this valuable input in an effective and efficient manner. Equally important is provision of support for clinical educators and mentors from clinical departments; protected time and acknowledgement of the importance of the role are both invaluable. The support offered to radiation therapy students by clinical staff is more highly valued than physical resources or models of placement support. Joint investment in mentor support by both universities and clinical departments is crucial for facilitation of effective clinical learning.

## Conflict of Interest

None declared.

## Funding Information

This study was not funded.

## References

[1] Doughty J, Hodgson D (2009). Evaluation of a new clinical support model in radiotherapy practice. Nurse Educ Pract.

[2] Newton JM, Billett S, Ockerby CM (2009). Journeying through clinical placements – An examination of six student cases. Nurse Educ Today.

[3] Gilmour C, McIntyre M, McLelland G, Hall H, Miles M (2013). Exploring the impact of clinical placement models on undergraduate midwifery students. Women Birth.

[4] Moreland N, Carnwell R (2000). Co-opting learners: Addressing the learning support needs through a learning support needs questionnaire. Part one: The rationale and basis of the learning support needs questionnaire. Res Post-Comp Educ.

[5] Price L, Hastie L, Duffy K, Ness V, McCallum J (2011). Supporting students in clinical practice: Pre-registration nursing students’ views on the role of the lecturer. Nurse Educ Today.

[6] Wessa P (2012). Pearson Correlation (v1.0.6) in Free Statistics Software (v1.1.23-r&).

[7] McCall L, Wray N, McKenna L (2009). Influence of clinical placement on undergraduate midwifery students’ career intentions. Midwifery.

[8] Ogbu SOI (2008). Radiography students’ perceptions of clinical placements – A Nigerian perspective. Radiography.

[9] Lambert V, Glacken M (2005). Clinical education facilitators: A literature review. J Clin Nurs.

[10] Myall M, Levett-Jones T, Lathlean J (2008). Mentorship in contemporary practice: The experiences of nursing students and practice mentors. J Clin Nurs.

[11] Dadge J, Casey D (2009). Supporting mentors in clinical practice. Paediatr Nurs.

[12] Nursing and Midwifery Council (2010). Standards for Pre-registration Nursing Education.

[13] Levett-Jones T, Fahy K, Parsons K, Mitchell A (2006). Enhancing nursing students’ clinical placement experiences. Contemp Nurse.

[14] Cantillon P, Sargeant J (2008). Giving feedback in clinical settings. BMJ.

[15] Jonsen E, Melender H, Hilli Y (2013). Finnish and Swedish nursing students’ experiences of their first clinical practice placement – A qualitative study. Nurse Educ Today.

[16] Veeramah V (2012). What are the barriers to good mentoring?. Nurs Pract.

[17] Mitchell G (2003). Nursing shortage or nursing famine: Looking beyond the numbers. Nurs Sci Q.

[18] O'Keefe M, Burgess T, McAllister S, Stupans I (2012). Twelve tips for supporting student learning in multidisciplinary clinical placements. Med Teach.

